# Supplementary material for: Short-term exposure to PM2.5 and 1.5 million deaths: a time-stratified case-crossover analysis in the Mexico City Metropolitan Area
Source: Environ Health. 2023 Oct 17;22:70. doi: 10.1186/s12940-023-01024-4 (PMC10580614; doi:10.1186/s12940-023-01024-4)
Supplement: Supplementary file 1 — Additional file 1: Figure S1. Cumulative percentage increase (%) and 95% CI for non-accidental mortality by education, insurance type and job categories (adults ≥18 years-old) per 10 μg/m3 increase in PM2.5 over two days (lag01) and one week (lag06) in the Mexico City Metropolitan Area for 2004-2019. Job category associations are for the period from 2013-2019. Table S1. Linearity tests in the associations between short-term exposure to PM2.5 and broad-group mortality outcomes. Table S2. Single lag and cumulative Odds Ratios and 95% confidence intervals for broad-group mortality outcomes associated with 10μg/m3 increase in PM2.5. Table S3. Single lag and cumulative Odds Ratios and 95% confidence intervals for cause-specific mortality outcomes associated with 10μg/m3 increase in PM2.5. [file 12940_2023_1024_MOESM1_ESM.docx]

**Supplemental material**

Figure S1. Cumulative percentage increase (%) and 95% CI for non-accidental mortality by education, insurance type and job categories (adults ≥18 years-old) per 10 μg/m^3^ increase in PM_2.5_ over two days (lag_01_) and one week (lag_06_) in the Mexico City Metropolitan Area for 2004-2019. Job category associations are for the period from 2013-2019.

Table S1. Linearity tests in the associations between short-term exposure to PM_2.5_ and broad-group mortality outcomes.

Table S2. Single lag and cumulative Odds Ratios and 95% confidence intervals for broad-group mortality outcomes associated with 10μg/m^3^ increase in PM_2.5_

Table S3. Single lag and cumulative Odds Ratios and 95% confidence intervals for cause-specific mortality outcomes associated with 10μg/m^3^ increase in PM_2.5_


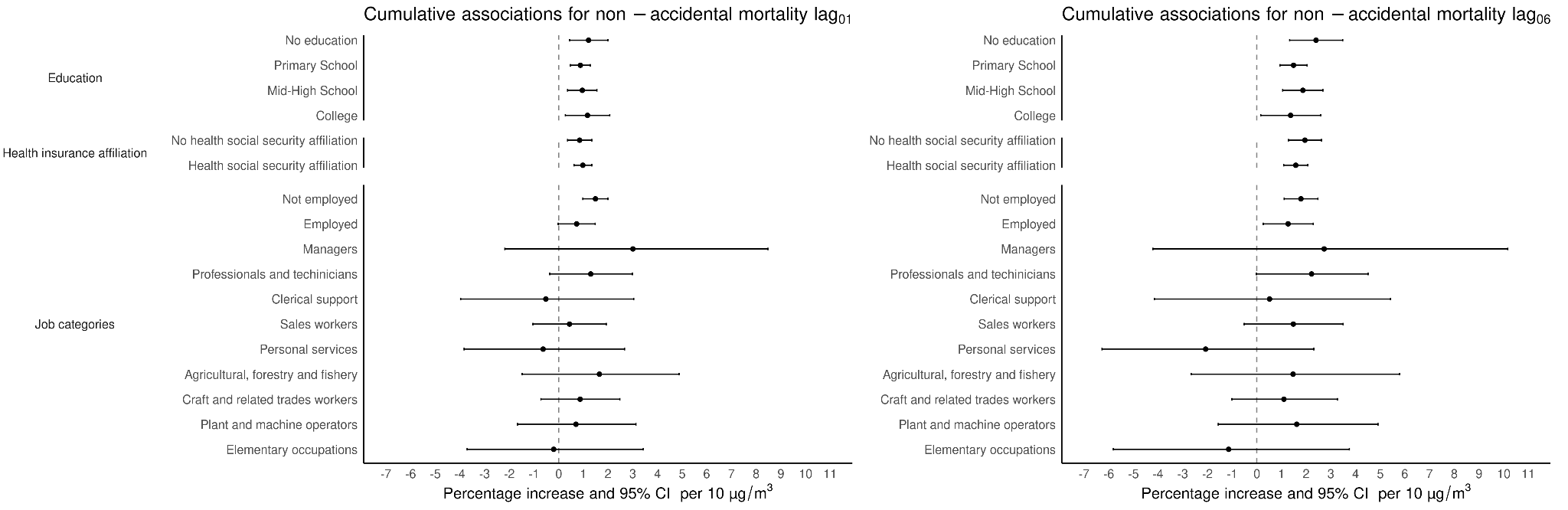


Figure S1. Cumulative percentage increase (%) and 95% CI for non-accidental mortality by education, health insurance affiliation, and job categories (adults ≥18 years-old) per 10 μg/m^3^ increase in PM_2.5_ over two days (lag_01_) and one week (lag_06_) in the Mexico City Metropolitan Area for 2004-2019. Job category associations are for the period from 2013-2019.

Table S1. Linearity tests in the associations between short-term exposure to PM_2.5_ and broad-group mortality outcomes.

| **ICD-10 Group** | **Model fit** | **Resid. Df** | **Resid. Dev** | **Df** | **Deviance** | ***p*-Value** |
| --- | --- | --- | --- | --- | --- | --- |
| Mental and behavioral (F00-F99) | linear | 2742.00 | 1835.89 |  |  |  |
|  | non-linear | 2741.24 | 1835.37 | 0.76 | 0.53 | 0.37 |
| Nervous system (G00-G99) | linear | 6810.19 | 4559.77 |  |  |  |
|  | non-linear | 6810.17 | 4559.76 | 0.02 | 0.01 | 0.04 |
| Circulatory system (I00-I99) | linear | 127908.40 | 85770.76 |  |  |  |
|  | non-linear | 127902.68 | 85764.87 | 5.72 | 5.89 | 0.40 |
| Respiratory system (J00-J99) | linear | 42209.22 | 28327.66 |  |  |  |
|  | non-linear | 42198.99 | 28309.93 | 10.23 | 17.72 | 0.07 |
| Digestive system (K00-K93) | linear | 51118.95 | 34286.18 |  |  |  |
|  | non-linear | 51112.74 | 34279.36 | 6.21 | 6.82 | 0.36 |
| Genitourinary (N00-N99) | linear | 16836.97 | 11291.25 |  |  |  |
|  | non-linear | 16829.15 | 11276.68 | 7.83 | 14.57 | 0.06 |
| Intentional self-harm (X60-X84) | linear | 2953.99 | 1986.60 |  |  |  |
|  | non-linear | 2950.25 | 1982.27 | 3.74 | 4.33 | 0.33 |

"Resid. Df" means degrees of freedom remaining after fitting linear PM_2.5_ terms (against the simplest model) and non-linear PM_2.5_ terms (against the linear model).

"Resid. Dev" means deviance remaining after fitting linear PM_2.5_ terms (against the simplest model) and non-linear PM_2.5_ terms (against the linear model).

"Df" means degrees of freedom attributed to inclusion of non-linear PM_2.5_ terms in our models.

"Deviance" means deviance reduction attributed to inclusion of non-linear PM_2.5_ terms in our models.

*p*-Values are based on likelihood ratio test comparisons.

Table S2. Single lag and cumulative Odds Ratios and 95% confidence intervals for broad-category mortality outcomes associated with 10μg/m^3^ increase in PM_2.5_

| **Broad-category mortality outcome** | **lag** | **OR** | **Lower Limit** | **Upper Limit** | **OR cumulative** | **Lower Limit cumulative** | **Upper Limit cumulative** |
| --- | --- | --- | --- | --- | --- | --- | --- |
| All non-accidental adults (18-64 years-old) | 0 | 1.008 | 1.004 | 1.012 | 1.008 | 1.004 | 1.012 |
|  | 1 | 0.999 | 0.994 | 1.004 | 1.007 | 1.003 | 1.012 |
|  | 2 | 1.004 | 0.999 | 1.008 | 1.011 | 1.006 | 1.016 |
|  | 3 | 1.002 | 0.997 | 1.006 | 1.012 | 1.007 | 1.018 |
|  | 4 | 0.998 | 0.993 | 1.002 | 1.010 | 1.004 | 1.016 |
|  | 5 | 1.002 | 0.997 | 1.006 | 1.012 | 1.005 | 1.018 |
|  | 6 | 1.005 | 1.001 | 1.009 | 1.017 | 1.010 | 1.023 |
| All non-accidental elderly (+65 years-old) | 0 | 1.005 | 1.002 | 1.008 | 1.005 | 1.002 | 1.008 |
|  | 1 | 1.005 | 1.002 | 1.009 | 1.010 | 1.007 | 1.014 |
|  | 2 | 0.997 | 0.993 | 1.001 | 1.007 | 1.003 | 1.011 |
|  | 3 | 1.001 | 0.997 | 1.004 | 1.008 | 1.004 | 1.012 |
|  | 4 | 1.003 | 0.999 | 1.006 | 1.011 | 1.006 | 1.015 |
|  | 5 | 1.002 | 0.998 | 1.005 | 1.013 | 1.008 | 1.018 |
|  | 6 | 1.004 | 1.001 | 1.007 | 1.017 | 1.012 | 1.022 |
| All non-accidental Men | 0 | 1.007 | 1.003 | 1.010 | 1.007 | 1.003 | 1.010 |
|  | 1 | 1.002 | 0.998 | 1.006 | 1.008 | 1.004 | 1.012 |
|  | 2 | 1.000 | 0.996 | 1.004 | 1.008 | 1.004 | 1.012 |
|  | 3 | 1.000 | 0.996 | 1.004 | 1.008 | 1.003 | 1.012 |
|  | 4 | 1.000 | 0.996 | 1.004 | 1.008 | 1.003 | 1.013 |
|  | 5 | 1.004 | 1.000 | 1.008 | 1.012 | 1.006 | 1.017 |
|  | 6 | 1.006 | 1.002 | 1.009 | 1.017 | 1.012 | 1.023 |
| All non-accidental Women | 0 | 1.006 | 1.002 | 1.010 | 1.006 | 1.002 | 1.010 |
|  | 1 | 1.004 | 1.000 | 1.008 | 1.010 | 1.006 | 1.014 |
|  | 2 | 0.999 | 0.995 | 1.003 | 1.009 | 1.005 | 1.014 |
|  | 3 | 1.002 | 0.998 | 1.006 | 1.012 | 1.007 | 1.016 |
|  | 4 | 1.002 | 0.998 | 1.006 | 1.014 | 1.008 | 1.019 |
|  | 5 | 0.999 | 0.995 | 1.003 | 1.013 | 1.007 | 1.018 |
|  | 6 | 1.003 | 0.999 | 1.007 | 1.016 | 1.010 | 1.021 |
| All Mental and behavioral disorders adults | 0 | 0.983 | 0.942 | 1.026 | 0.983 | 0.942 | 1.026 |
|  | 1 | 0.998 | 0.952 | 1.047 | 0.981 | 0.935 | 1.029 |
|  | 2 | 0.982 | 0.936 | 1.029 | 0.963 | 0.914 | 1.014 |
|  | 3 | 0.955 | 0.910 | 1.003 | 0.920 | 0.870 | 0.973 |
|  | 4 | 1.022 | 0.976 | 1.071 | 0.940 | 0.886 | 0.998 |
|  | 5 | 0.995 | 0.950 | 1.043 | 0.936 | 0.879 | 0.997 |
|  | 6 | 1.015 | 0.975 | 1.057 | 0.950 | 0.891 | 1.013 |
| All Mental and behavioral disorders elderly | 0 | 1.038 | 0.990 | 1.089 | 1.038 | 0.990 | 1.089 |
|  | 1 | 0.997 | 0.946 | 1.051 | 1.035 | 0.982 | 1.092 |
|  | 2 | 1.016 | 0.962 | 1.073 | 1.052 | 0.991 | 1.115 |
|  | 3 | 1.023 | 0.970 | 1.079 | 1.076 | 1.010 | 1.146 |
|  | 4 | 1.008 | 0.955 | 1.063 | 1.084 | 1.013 | 1.160 |
|  | 5 | 0.966 | 0.914 | 1.021 | 1.047 | 0.975 | 1.125 |
|  | 6 | 0.989 | 0.942 | 1.039 | 1.036 | 0.963 | 1.114 |
| All nervous adults | 0 | 1.029 | 1.001 | 1.058 | 1.029 | 1.001 | 1.058 |
|  | 1 | 0.978 | 0.948 | 1.009 | 1.007 | 0.976 | 1.039 |
|  | 2 | 1.013 | 0.981 | 1.046 | 1.020 | 0.985 | 1.056 |
|  | 3 | 0.975 | 0.945 | 1.007 | 0.995 | 0.958 | 1.032 |
|  | 4 | 0.983 | 0.953 | 1.014 | 0.977 | 0.940 | 1.016 |
|  | 5 | 1.001 | 0.971 | 1.033 | 0.979 | 0.939 | 1.020 |
|  | 6 | 0.998 | 0.971 | 1.027 | 0.977 | 0.937 | 1.019 |
| All nervous elderly | 0 | 1.002 | 0.973 | 1.032 | 1.002 | 0.973 | 1.032 |
|  | 1 | 1.022 | 0.988 | 1.056 | 1.023 | 0.990 | 1.058 |
|  | 2 | 0.993 | 0.960 | 1.026 | 1.016 | 0.979 | 1.053 |
|  | 3 | 0.980 | 0.948 | 1.014 | 0.996 | 0.957 | 1.035 |
|  | 4 | 1.023 | 0.989 | 1.058 | 1.018 | 0.977 | 1.061 |
|  | 5 | 0.992 | 0.959 | 1.025 | 1.010 | 0.967 | 1.055 |
|  | 6 | 0.977 | 0.949 | 1.006 | 0.987 | 0.944 | 1.032 |
| All cardiovascular adults | 0 | 1.020 | 1.010 | 1.030 | 1.020 | 1.010 | 1.030 |
|  | 1 | 0.990 | 0.980 | 1.001 | 1.010 | 1.000 | 1.021 |
|  | 2 | 1.004 | 0.993 | 1.014 | 1.014 | 1.002 | 1.026 |
|  | 3 | 0.996 | 0.985 | 1.006 | 1.009 | 0.997 | 1.022 |
|  | 4 | 0.996 | 0.985 | 1.006 | 1.005 | 0.991 | 1.018 |
|  | 5 | 1.000 | 0.989 | 1.010 | 1.004 | 0.991 | 1.019 |
|  | 6 | 1.009 | 1.000 | 1.019 | 1.014 | 1.000 | 1.028 |
| All cardiovascular elderly | 0 | 1.004 | 0.999 | 1.010 | 1.004 | 0.999 | 1.010 |
|  | 1 | 1.006 | 1.000 | 1.012 | 1.010 | 1.004 | 1.016 |
|  | 2 | 0.998 | 0.992 | 1.004 | 1.008 | 1.002 | 1.015 |
|  | 3 | 1.000 | 0.994 | 1.006 | 1.008 | 1.001 | 1.015 |
|  | 4 | 1.007 | 1.001 | 1.013 | 1.015 | 1.007 | 1.023 |
|  | 5 | 1.000 | 0.994 | 1.006 | 1.015 | 1.007 | 1.024 |
|  | 6 | 1.003 | 0.998 | 1.008 | 1.018 | 1.010 | 1.027 |
| All cerebrovascular adults | 0 | 1.032 | 1.012 | 1.053 | 1.032 | 1.012 | 1.053 |
|  | 1 | 0.996 | 0.974 | 1.019 | 1.028 | 1.005 | 1.052 |
|  | 2 | 0.980 | 0.958 | 1.003 | 1.008 | 0.983 | 1.034 |
|  | 3 | 1.013 | 0.991 | 1.036 | 1.022 | 0.994 | 1.050 |
|  | 4 | 0.997 | 0.974 | 1.020 | 1.019 | 0.990 | 1.048 |
|  | 5 | 1.004 | 0.981 | 1.027 | 1.022 | 0.992 | 1.054 |
|  | 6 | 1.014 | 0.993 | 1.035 | 1.036 | 1.005 | 1.069 |
| All cerebrovascular elderly | 0 | 0.994 | 0.982 | 1.006 | 0.994 | 0.982 | 1.006 |
|  | 1 | 1.003 | 0.990 | 1.017 | 0.997 | 0.984 | 1.011 |
|  | 2 | 1.000 | 0.987 | 1.014 | 0.998 | 0.983 | 1.013 |
|  | 3 | 0.999 | 0.985 | 1.012 | 0.996 | 0.981 | 1.012 |
|  | 4 | 0.999 | 0.986 | 1.013 | 0.996 | 0.979 | 1.013 |
|  | 5 | 1.006 | 0.992 | 1.019 | 1.001 | 0.983 | 1.019 |
|  | 6 | 1.005 | 0.993 | 1.017 | 1.006 | 0.988 | 1.024 |
| All respiratory adults | 0 | 1.009 | 0.992 | 1.025 | 1.009 | 0.992 | 1.025 |
|  | 1 | 1.016 | 0.998 | 1.035 | 1.025 | 1.006 | 1.044 |
|  | 2 | 0.988 | 0.970 | 1.006 | 1.013 | 0.992 | 1.034 |
|  | 3 | 1.005 | 0.987 | 1.024 | 1.018 | 0.996 | 1.041 |
|  | 4 | 1.003 | 0.985 | 1.021 | 1.021 | 0.997 | 1.045 |
|  | 5 | 0.993 | 0.975 | 1.012 | 1.014 | 0.989 | 1.039 |
|  | 6 | 1.012 | 0.995 | 1.029 | 1.026 | 1.000 | 1.052 |
| All respiratory elderly | 0 | 1.002 | 0.993 | 1.011 | 1.002 | 0.993 | 1.011 |
|  | 1 | 1.018 | 1.008 | 1.028 | 1.020 | 1.010 | 1.030 |
|  | 2 | 0.993 | 0.983 | 1.003 | 1.013 | 1.002 | 1.024 |
|  | 3 | 1.004 | 0.994 | 1.014 | 1.017 | 1.005 | 1.029 |
|  | 4 | 0.996 | 0.986 | 1.006 | 1.013 | 1.000 | 1.026 |
|  | 5 | 1.002 | 0.992 | 1.012 | 1.015 | 1.001 | 1.029 |
|  | 6 | 1.019 | 1.010 | 1.028 | 1.035 | 1.020 | 1.049 |
| All digestive adults | 0 | 1.008 | 0.998 | 1.019 | 1.008 | 0.998 | 1.019 |
|  | 1 | 0.997 | 0.985 | 1.009 | 1.006 | 0.994 | 1.017 |
|  | 2 | 1.016 | 1.004 | 1.028 | 1.022 | 1.008 | 1.035 |
|  | 3 | 0.998 | 0.986 | 1.010 | 1.019 | 1.005 | 1.034 |
|  | 4 | 0.999 | 0.987 | 1.011 | 1.018 | 1.003 | 1.034 |
|  | 5 | 1.006 | 0.994 | 1.018 | 1.024 | 1.009 | 1.041 |
|  | 6 | 0.996 | 0.986 | 1.007 | 1.021 | 1.005 | 1.037 |
| All digestive elderly | 0 | 1.004 | 0.994 | 1.015 | 1.004 | 0.994 | 1.015 |
|  | 1 | 1.008 | 0.996 | 1.020 | 1.012 | 1.000 | 1.024 |
|  | 2 | 0.994 | 0.982 | 1.006 | 1.005 | 0.992 | 1.018 |
|  | 3 | 0.996 | 0.984 | 1.008 | 1.001 | 0.988 | 1.015 |
|  | 4 | 1.004 | 0.992 | 1.016 | 1.005 | 0.991 | 1.020 |
|  | 5 | 0.994 | 0.983 | 1.006 | 1.000 | 0.984 | 1.015 |
|  | 6 | 1.008 | 0.998 | 1.019 | 1.008 | 0.992 | 1.024 |
| All genitourinary adults | 0 | 0.996 | 0.975 | 1.017 | 0.996 | 0.975 | 1.017 |
|  | 1 | 0.996 | 0.972 | 1.020 | 0.992 | 0.969 | 1.015 |
|  | 2 | 0.985 | 0.962 | 1.009 | 0.977 | 0.952 | 1.003 |
|  | 3 | 1.047 | 1.023 | 1.072 | 1.023 | 0.995 | 1.052 |
|  | 4 | 1.001 | 0.978 | 1.025 | 1.024 | 0.994 | 1.054 |
|  | 5 | 0.996 | 0.972 | 1.020 | 1.019 | 0.988 | 1.052 |
|  | 6 | 1.014 | 0.993 | 1.036 | 1.034 | 1.001 | 1.067 |
| All genitourinary elderly | 0 | 1.005 | 0.988 | 1.021 | 1.005 | 0.988 | 1.021 |
|  | 1 | 1.007 | 0.988 | 1.025 | 1.011 | 0.993 | 1.030 |
|  | 2 | 1.004 | 0.985 | 1.022 | 1.015 | 0.995 | 1.036 |
|  | 3 | 0.999 | 0.981 | 1.018 | 1.014 | 0.992 | 1.036 |
|  | 4 | 0.998 | 0.980 | 1.017 | 1.012 | 0.990 | 1.036 |
|  | 5 | 1.011 | 0.993 | 1.030 | 1.024 | 0.999 | 1.048 |
|  | 6 | 0.999 | 0.983 | 1.016 | 1.023 | 0.998 | 1.048 |

Table S3. Single lag and cumulative Odds Ratios and 95% confidence intervals for cause-specific mortality outcomes associated with 10μg/m^3^ increase in PM_2.5_

| **Cause-specific mortality outcome** | **lag** | **OR** | **Lower Limit** | **Upper Limit** | **OR cumulative** | **Lower Limit cumulative** | **Upper Limit cumulative** |
| --- | --- | --- | --- | --- | --- | --- | --- |
| Extrapyramidal and movement disorders | 0 | 0.991 | 0.942 | 1.043 | 0.991 | 0.942 | 1.043 |
|  | 1 | 1.048 | 0.988 | 1.111 | 1.039 | 0.980 | 1.101 |
|  | 2 | 0.966 | 0.910 | 1.025 | 1.003 | 0.941 | 1.070 |
|  | 3 | 0.991 | 0.936 | 1.049 | 0.994 | 0.928 | 1.064 |
|  | 4 | 1.027 | 0.970 | 1.087 | 1.020 | 0.949 | 1.096 |
|  | 5 | 0.976 | 0.922 | 1.034 | 0.996 | 0.922 | 1.075 |
|  | 6 | 1.000 | 0.950 | 1.053 | 0.996 | 0.921 | 1.076 |
| Chronic rheumatic heart disease | 0 | 0.978 | 0.932 | 1.026 | 0.978 | 0.932 | 1.026 |
|  | 1 | 1.035 | 0.982 | 1.091 | 1.012 | 0.960 | 1.067 |
|  | 2 | 1.013 | 0.964 | 1.065 | 1.025 | 0.968 | 1.085 |
|  | 3 | 1.014 | 0.964 | 1.067 | 1.039 | 0.976 | 1.106 |
|  | 4 | 1.021 | 0.971 | 1.075 | 1.061 | 0.993 | 1.135 |
|  | 5 | 0.999 | 0.950 | 1.051 | 1.060 | 0.989 | 1.138 |
|  | 6 | 0.948 | 0.905 | 0.992 | 1.005 | 0.935 | 1.080 |
| Hypertensive diseases | 0 | 1.013 | 1.000 | 1.027 | 1.013 | 1.000 | 1.027 |
|  | 1 | 0.998 | 0.984 | 1.013 | 1.011 | 0.997 | 1.026 |
|  | 2 | 1.002 | 0.987 | 1.016 | 1.013 | 0.997 | 1.029 |
|  | 3 | 1.006 | 0.992 | 1.021 | 1.019 | 1.002 | 1.037 |
|  | 4 | 0.997 | 0.982 | 1.012 | 1.016 | 0.998 | 1.035 |
|  | 5 | 1.001 | 0.987 | 1.016 | 1.018 | 0.998 | 1.038 |
|  | 6 | 1.005 | 0.992 | 1.018 | 1.023 | 1.003 | 1.043 |
| Acute ischemic heart disease | 0 | 1.006 | 1.000 | 1.013 | 1.006 | 1.000 | 1.013 |
|  | 1 | 1.006 | 0.998 | 1.013 | 1.012 | 1.005 | 1.020 |
|  | 2 | 0.999 | 0.992 | 1.007 | 1.011 | 1.003 | 1.020 |
|  | 3 | 0.997 | 0.989 | 1.004 | 1.008 | 0.999 | 1.017 |
|  | 4 | 1.005 | 0.997 | 1.012 | 1.013 | 1.004 | 1.023 |
|  | 5 | 0.999 | 0.992 | 1.006 | 1.012 | 1.002 | 1.022 |
|  | 6 | 1.004 | 0.998 | 1.011 | 1.016 | 1.006 | 1.026 |
| Chronic ischemic heart disease | 0 | 1.023 | 1.002 | 1.044 | 1.023 | 1.002 | 1.044 |
|  | 1 | 0.992 | 0.969 | 1.016 | 1.015 | 0.992 | 1.039 |
|  | 2 | 1.012 | 0.988 | 1.036 | 1.027 | 1.000 | 1.054 |
|  | 3 | 0.973 | 0.950 | 0.996 | 0.999 | 0.971 | 1.027 |
|  | 4 | 1.037 | 1.014 | 1.062 | 1.036 | 1.006 | 1.067 |
|  | 5 | 0.984 | 0.962 | 1.007 | 1.020 | 0.988 | 1.052 |
|  | 6 | 1.004 | 0.984 | 1.025 | 1.024 | 0.992 | 1.058 |
| Pulmonary heart disease | 0 | 1.021 | 0.985 | 1.059 | 1.021 | 0.985 | 1.059 |
|  | 1 | 0.998 | 0.958 | 1.040 | 1.019 | 0.978 | 1.062 |
|  | 2 | 0.998 | 0.958 | 1.040 | 1.018 | 0.972 | 1.065 |
|  | 3 | 1.004 | 0.964 | 1.045 | 1.021 | 0.972 | 1.073 |
|  | 4 | 0.994 | 0.953 | 1.037 | 1.015 | 0.963 | 1.070 |
|  | 5 | 1.000 | 0.960 | 1.042 | 1.015 | 0.960 | 1.073 |
|  | 6 | 0.999 | 0.963 | 1.036 | 1.014 | 0.958 | 1.072 |
| Other forms of heart disease | 0 | 1.006 | 0.988 | 1.024 | 1.006 | 0.988 | 1.024 |
|  | 1 | 0.999 | 0.979 | 1.019 | 1.005 | 0.985 | 1.025 |
|  | 2 | 1.004 | 0.984 | 1.024 | 1.008 | 0.987 | 1.031 |
|  | 3 | 1.004 | 0.985 | 1.024 | 1.013 | 0.989 | 1.037 |
|  | 4 | 0.997 | 0.978 | 1.017 | 1.010 | 0.985 | 1.035 |
|  | 5 | 1.011 | 0.991 | 1.031 | 1.021 | 0.994 | 1.048 |
|  | 6 | 1.003 | 0.985 | 1.021 | 1.024 | 0.997 | 1.052 |
| Stroke hemorrhagic | 0 | 1.030 | 1.011 | 1.049 | 1.030 | 1.011 | 1.049 |
|  | 1 | 0.983 | 0.963 | 1.003 | 1.012 | 0.992 | 1.033 |
|  | 2 | 1.012 | 0.991 | 1.033 | 1.024 | 1.001 | 1.048 |
|  | 3 | 1.009 | 0.989 | 1.030 | 1.034 | 1.009 | 1.059 |
|  | 4 | 0.987 | 0.967 | 1.008 | 1.021 | 0.994 | 1.047 |
|  | 5 | 1.006 | 0.986 | 1.028 | 1.027 | 0.999 | 1.056 |
|  | 6 | 1.009 | 0.991 | 1.028 | 1.036 | 1.008 | 1.065 |
| Stroke ischemic | 0 | 1.005 | 0.976 | 1.035 | 1.005 | 0.976 | 1.035 |
|  | 1 | 1.021 | 0.988 | 1.055 | 1.026 | 0.993 | 1.060 |
|  | 2 | 1.005 | 0.972 | 1.039 | 1.031 | 0.994 | 1.069 |
|  | 3 | 0.992 | 0.960 | 1.026 | 1.023 | 0.984 | 1.063 |
|  | 4 | 1.031 | 0.998 | 1.065 | 1.055 | 1.012 | 1.099 |
|  | 5 | 0.984 | 0.952 | 1.017 | 1.038 | 0.993 | 1.084 |
|  | 6 | 0.999 | 0.970 | 1.029 | 1.037 | 0.992 | 1.083 |
| Diseases of arteries | 0 | 1.046 | 1.007 | 1.087 | 1.046 | 1.007 | 1.087 |
|  | 1 | 0.973 | 0.931 | 1.017 | 1.018 | 0.975 | 1.063 |
|  | 2 | 0.960 | 0.919 | 1.003 | 0.978 | 0.932 | 1.025 |
|  | 3 | 1.001 | 0.959 | 1.044 | 0.978 | 0.930 | 1.030 |
|  | 4 | 1.033 | 0.990 | 1.077 | 1.010 | 0.957 | 1.067 |
|  | 5 | 0.944 | 0.903 | 0.987 | 0.954 | 0.900 | 1.012 |
|  | 6 | 1.032 | 0.991 | 1.074 | 0.984 | 0.928 | 1.044 |
| Influenza and pneumonia | 0 | 1.019 | 1.006 | 1.032 | 1.019 | 1.006 | 1.032 |
|  | 1 | 1.011 | 0.996 | 1.026 | 1.030 | 1.015 | 1.045 |
|  | 2 | 0.997 | 0.983 | 1.012 | 1.027 | 1.010 | 1.044 |
|  | 3 | 1.004 | 0.989 | 1.019 | 1.031 | 1.013 | 1.049 |
|  | 4 | 0.996 | 0.981 | 1.010 | 1.027 | 1.008 | 1.046 |
|  | 5 | 1.000 | 0.986 | 1.014 | 1.027 | 1.007 | 1.047 |
|  | 6 | 1.022 | 1.009 | 1.035 | 1.049 | 1.028 | 1.070 |
| Chronic respiratory disease | 0 | 0.998 | 0.987 | 1.009 | 0.998 | 0.987 | 1.009 |
|  | 1 | 1.017 | 1.004 | 1.030 | 1.015 | 1.002 | 1.028 |
|  | 2 | 0.995 | 0.983 | 1.008 | 1.010 | 0.996 | 1.025 |
|  | 3 | 1.008 | 0.996 | 1.022 | 1.019 | 1.003 | 1.034 |
|  | 4 | 0.994 | 0.981 | 1.006 | 1.012 | 0.996 | 1.029 |
|  | 5 | 1.001 | 0.988 | 1.014 | 1.013 | 0.996 | 1.030 |
|  | 6 | 1.012 | 1.000 | 1.024 | 1.025 | 1.007 | 1.043 |
| Diseases of esophagus, stomach and duodenum | 0 | 0.987 | 0.960 | 1.014 | 0.987 | 0.960 | 1.014 |
|  | 1 | 1.034 | 1.003 | 1.066 | 1.020 | 0.989 | 1.052 |
|  | 2 | 0.997 | 0.967 | 1.028 | 1.017 | 0.983 | 1.053 |
|  | 3 | 0.990 | 0.959 | 1.022 | 1.007 | 0.971 | 1.045 |
|  | 4 | 0.995 | 0.964 | 1.026 | 1.002 | 0.963 | 1.042 |
|  | 5 | 1.009 | 0.978 | 1.041 | 1.011 | 0.970 | 1.054 |
|  | 6 | 0.996 | 0.968 | 1.024 | 1.007 | 0.965 | 1.050 |
| Diseases of liver | 0 | 1.003 | 0.993 | 1.013 | 1.003 | 0.993 | 1.013 |
|  | 1 | 1.000 | 0.989 | 1.012 | 1.003 | 0.992 | 1.014 |
|  | 2 | 1.006 | 0.995 | 1.017 | 1.009 | 0.997 | 1.022 |
|  | 3 | 0.995 | 0.984 | 1.006 | 1.004 | 0.991 | 1.018 |
|  | 4 | 1.003 | 0.992 | 1.014 | 1.007 | 0.993 | 1.022 |
|  | 5 | 1.008 | 0.997 | 1.019 | 1.015 | 1.000 | 1.031 |
|  | 6 | 1.003 | 0.993 | 1.013 | 1.018 | 1.003 | 1.034 |
| Disorders of gallbladder, biliary tract and pancreas | 0 | 1.012 | 0.987 | 1.038 | 1.012 | 0.987 | 1.038 |
|  | 1 | 0.995 | 0.967 | 1.024 | 1.007 | 0.980 | 1.036 |
|  | 2 | 1.020 | 0.992 | 1.049 | 1.027 | 0.996 | 1.059 |
|  | 3 | 0.992 | 0.964 | 1.021 | 1.019 | 0.986 | 1.053 |
|  | 4 | 1.008 | 0.980 | 1.036 | 1.027 | 0.992 | 1.063 |
|  | 5 | 0.989 | 0.962 | 1.018 | 1.016 | 0.979 | 1.054 |
|  | 6 | 1.000 | 0.975 | 1.025 | 1.016 | 0.979 | 1.054 |
| Renal failure | 0 | 1.001 | 0.983 | 1.018 | 1.001 | 0.983 | 1.018 |
|  | 1 | 1.005 | 0.986 | 1.025 | 1.006 | 0.986 | 1.025 |
|  | 2 | 0.987 | 0.968 | 1.006 | 0.992 | 0.971 | 1.013 |
|  | 3 | 1.014 | 0.994 | 1.034 | 1.006 | 0.983 | 1.029 |
|  | 4 | 1.008 | 0.988 | 1.028 | 1.013 | 0.989 | 1.039 |
|  | 5 | 1.009 | 0.990 | 1.029 | 1.023 | 0.997 | 1.050 |
|  | 6 | 1.012 | 0.994 | 1.030 | 1.035 | 1.008 | 1.062 |
| Suicide | 0 | 1.021 | 0.989 | 1.054 | 1.021 | 0.989 | 1.054 |
|  | 1 | 0.979 | 0.945 | 1.015 | 1.000 | 0.965 | 1.035 |
|  | 2 | 1.018 | 0.982 | 1.055 | 1.018 | 0.979 | 1.058 |
|  | 3 | 0.985 | 0.950 | 1.022 | 1.002 | 0.961 | 1.045 |
|  | 4 | 0.989 | 0.953 | 1.026 | 0.991 | 0.948 | 1.036 |
|  | 5 | 1.000 | 0.965 | 1.037 | 0.991 | 0.946 | 1.039 |
|  | 6 | 1.021 | 0.989 | 1.054 | 1.013 | 0.966 | 1.062 |
